# Urbanization Impact on Regional Sustainable Development: Through the Lens of Urban-Rural Resilience

**DOI:** 10.3390/ijerph192215407

**Published:** 2022-11-21

**Authors:** Chenchen Shi, Xiaoping Zhu, Haowei Wu, Zhihui Li

**Affiliations:** 1 School of Urban Economics and Public Administration, Capital University of Economics and Business, Beijing 100070, China; 2 Key Laboratory of Land Surface Pattern and Simulation, Institute of Geographic Sciences and Natural Resources Research, Chinese Academy of Sciences, Beijing 100101, China; 3 Beijing Key Laboratory of Megaregions Sustainable Development Modeling, Capital University of Economics and Business, Beijing 100070, China; 4 College of Agronomy and Biotechnology, Hebei Normal University of Science & Technology, Qinhuangdao 066104, China; 5 University of Chinese Academy of Sciences, Beijing 100049, China; 6 School of Soil and Water Conservation, Beijing Forestry University, Beijing 100083, China

**Keywords:** land use change, urbanization, urban–rural system, Beijing-Tianjin-Hebei urban agglomeration

## Abstract

The urban–rural system is an economically, socially, and environmentally interlinked space, which requires the integration of industry, space, and population. To achieve sustainable and coordinated development between urban and rural systems, dynamic land use change within the urban–rural system and the ecological and social consequences need to be clarified. This study uses system resilience to evaluate such an impact and explores the impact of land use change, especially land conversion induced by urbanization on regional development through the lens of urban–rural resilience. The empirical case is based on the Beijing-Tianjin-Hebei Urban Agglomeration (BTHUA) in China from 2000 to 2020 when there was rapid urbanization in this region. The results show that along with urbanization in the BTHUA, urban–rural resilience is high in urban core areas and low in peripheral areas. From the urban core to the rural outskirts, there is a general trend that comprehensive resilience decreases with decreased social resilience and increased ecological resilience in this region. Specifically, at the city level, comprehensive resilience decreases sharply from the urban center to its 3–5 km buffer zone and then remains relatively stable in the rural regions. A similar trend goes for social resilience at the city level, while ecological resilience increases sharply from the urban center to its 1–3 km buffer zone, and then remains relatively stable in the rural regions in this region, except for cities in the west and south of Hebei. This study contributes to the conceptualization and measurement of urban–rural resilience in the urban–rural system with empirical findings revealing the impact of rapid urbanization on urban–rural resilience over the last twenty years in the BTHUA in China. In addition, the spatial heterogeneity results could be used for policy reference to make targeted resilience strategies in the study region.

## 1. Introduction

Cities, which only came into being six thousand years ago, are young compared to the millions of years of humankind. More than half of the world’s population live in cities now, and the proportion is higher in developed countries; by 2050, about 68% of the world’s population are projected to live in cities [1]. However, along with urbanization, cities face problems, including the contradiction between the rapidly growing population and resources provision, air pollution, traffic congestion, inadequate public services, and urban diseases. Assessing urbanization’s ecological and social consequences is essential to future sustainable development. There are various approaches to examining sustainability in socioecological systems, such as Turner’s vulnerability approach [2], Ostrom’s diagnostic approach [3], Anderies’ robustness and coupled infrastructure approach [4], Liu’s telecoupling approach [5], and Wu’s landscape sustainability approach [6], while this research uses resilience to assess sustainability, as it is a powerful tool in characterizing the sustainability decision-making context [7]. The ecological and social effects of urbanization can be measured by urban resilience, the ability of an urban system to maintain its status quo or recover quickly to the desired level in the face of short-term shocks or long-term stresses, to adapt to changes, and to alter functions that limit current or future adaptive capacity [8]. Cities need to enhance their resilience to resolve the crisis brought on by urbanization. Suppose urbanization is compared to increasing the bones of a city by changing its structure (industrial structure, land use structure, and population structure). In that case, resilience is strengthening the muscles of the city to make it a healthy body/system.

In terms of the scale of resilience research, previous studies focus on resilience at the national level, urban clusters, cities, and even rural areas. The connotations and mechanisms of resilience are also different in different scales or realms. The concept of resilience first appeared in mechanical engineering, where engineering resilience refers to the ability of a system to return to its original state. Hence, when the studied system or system boundaries are different, so are the resilience connotations. While urban resilience focuses on cities as systems to enhance their social, economic, ecological, infrastructural, and governance capabilities, etc., against future risks [9], rural resilience focuses on rural regions. It enhances their abilities to adapt to uncertainties in the agricultural, forestry, and landscape services [10]. Though both urban and rural systems are complex socioecological systems [11], urban resilience pays more attention to the social function, while rural resilience concentrates on ecological function and economic independence [12]. In addition, the difference in resilience practice at the community level further leads to urban–rural resilience disparity [13]. However, resilience in an urban system cannot be improved without the support of the surrounding rural areas, and rural resilience and urban resilience have a lot in common. In addition, as the countryside evolves, some rural settlements are beginning to follow the urban development pattern. After all, the term “urban” in “urbanization” refers to not only cities but also townships, where rural residents live. Therefore, this study takes urban and rural areas as an integrated system and investigates resilience in the urban–rural continuum, especially within the metropolitan landscape [14].

The concept of urban–rural resilience [15] focuses on the urban–rural dependencies and partnerships to enhance resilience [16] and also suits the need for urban–rural integration. Land use data are used in this study to represent the urban–rural system [17]. This study uses grid-scale data to examine resilience across heterogeneous and uneven spaces with GIS as the spatial analyst and visualization tool. This does not mean that we do not acknowledge the urban–rural divide in resilience. Previous empirical research in the European Union, for example, observed the urban–rural hierarchy in resilience positively related to migration and that the resilience of intermediate and rural regions is contributed by agriculture [18]. Another piece of survey-based disaster resilience research in Nepal found that rural residents are more resilient in the post-earthquake relief progress [13]. Through this research, we aim to explore resilience change in the urban–rural system under a coherent framework.

Qualitative and quantitative tools are used to measure resilience on different scales. Qualitative measures explore the components of resilience through surveys or interviews [19,20]. At the same time, quantitative measures are used for the numerical analysis of resilience elements. Current quantitative measures of resilience mainly include indicator evaluation [9,21,22,23], resilience time function evaluation [24,25,26,27], and model simulation [28,29,30,31], among which the indicator evaluation of resilience is more common in resilience evaluation and can be divided into different subcategories according to the number of indicators [22,23], evaluation scale [9,21], and evaluated elements [22,32,33,34]. In this study, we adopt and further develop the evaluation index by Shi et al. [35] that assesses urban ecological resilience by delineating system sensitivity and adaptability. The advantage of Shi et al.’s method is that calculating the change rate of the resilience surrogate captures the critical mechanisms of resistance and recovery in resilience and characterizes threshold-crossing, which is a crucial resilience process [36]. In addition, previous urban agglomeration resilience assessments were often carried out at the administrative district level, which could not reflect the urban–rural distinction [37,38]. In contrast, this research is conducted on a grid scale to characterize urban–rural disparity.

Therefore, this study aims to quantitatively measure the resilience of the urban–rural system with the empirical case of the Beijing-Tianjin-Hebei region in China and to analyze the impact of urbanization on the resilience of the urban–rural system to propose strategies for urban–rural integration and resilience enhancement. While this section introduces the research background, relevant literature, and research aim, the following sections of this paper are structured as follows. Section 2 presents the case study, data source, and method this study uses to explore urbanization’s impact on urban–rural resilience. Section 3 presents the resilience evaluation results in the study region. Section 4 further analyzes urbanization (land use change)’s impact on the urban–rural system’s resilience. Section 5 concludes this research with a discussion of its theoretical, methodological, and empirical contributions, as well as possible limitations and future research agendas.

## 2. Materials and Methods

### 2.1. Case Study

**Figure 1:**
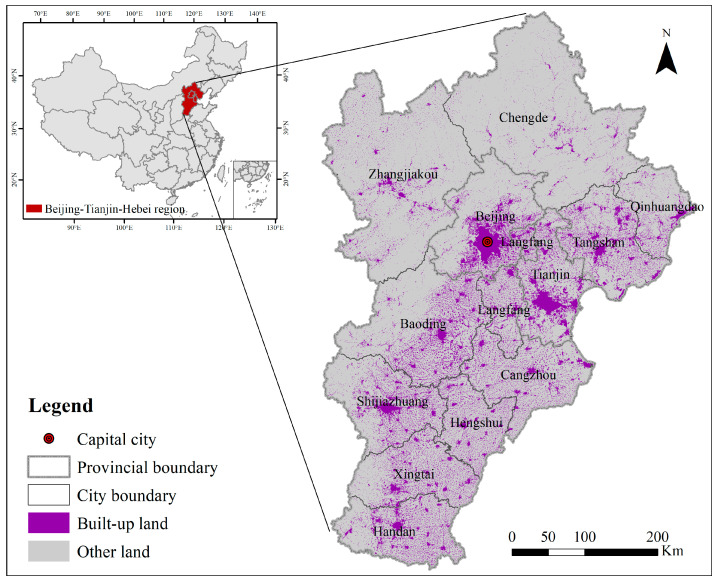
Location, built-up land, and administrative city boundaries of the BTHUA.

This study selects the Beijing-Tianjin-Hebei Urban Agglomeration (BTHUA) in China as the case study to empirically assess the impact of land use change on the resilience of the urban–rural system (Figure 1). As one of the major urban agglomerations in China, BTHUA has been a pilot of urbanization and sustainable development and is, hence, a good case for this study. In addition, the empirics of BTHUA can provide practical references for other urban agglomerations in China. The study period is set from 2000 to 2020 to observe urbanization and its social and ecological consequence in the past twenty years when China was experiencing rapid urbanization and a new-type urbanization period [39]. During the study period, in 2002, the 16th Party Congress promoted the continuous and rapid improvement of China’s urbanization level, and the urbanization rate also increased significantly. At the end of 2011, the urbanization rate of the resident population reached 51.27%, up 22.23 percentage points from the end of 1995, with an average annual increase of 1.39 percentage points [40].

In 2012, the 18th Party Congress proposed to take a new-type urbanization with Chinese characteristics [39], and the urbanization process entered a new stage of people-oriented and quality improvement. In recent years, to actively promote the construction of a new-type urbanization, supporting reforms in household registration, land, finance, education, employment, medical care, pension, and housing security has been promoted, and the urbanization rate has accelerated. From the end of 2011 to the end of 2017, the urbanization rate of the resident population increased by 7.25 percentage points, with an average annual increase of 1.21 percentage points [40]. At the end of 2017, the urbanization rate of the household population reached 42.35% [40]. In addition to rapid urban development, another reason we choose China as the study site is the long-lasting urban–rural divide in China caused by various reasons, including the rigid household registration system, opportunity inequality, uneven distribution of resources, and rent-seeking deeply rooted in the urban–rural duality in China. This study aims to assess the urban–rural divide from the resilience perspective.

**Figure 2:**
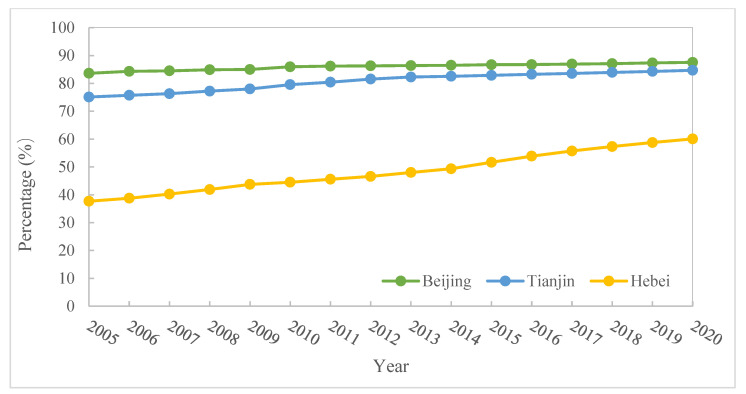
Urban population percentage in Beijing, Tianjin, and Hebei from 2005 to 2020. Note: The data are available from the China Population Statistics Yearbook since 2005.

The BTHUA is in the northern part of the North China Plain, with a total area of 216,000 km^2^, accounting for about 2.3% of the country’s total land area, and is one of the 19 national urban clusters under the new-type urbanization plannings of China [40]. The urbanization rate in this region was 66.7% in 2019 [41]. However, though under a coordinated development plan, this region’s urban–rural and intra-region gaps are still significant, manifested in the developed cities of Beijing and Tianjin and backward rural areas in Hebei (Figure 2). As for resilience in this region, according to resilience evaluation in previous research, the BTHUA is high in resilience compared with other areas of China, while within this region, the resilience ranking is Beijing, Tianjin, and Hebei, from high to low [42,43,44]. In the latest urban master plan (2016–2035), Beijing is dedicated to improving resilience to raise ecological quality, achieving sustainable use of water resources, and strengthening disaster prevention and mitigation capabilities. Hebei is devoted to building safe and resilient cities [45,46]. 

### 2.2. Data Source

This research uses Net Primary Production (NPP) and population data to quantify resilience in the urban–rural system. Land use data are used to represent urbanization and the urban–rural system. The NPP data (MOD17A3) of 1 km resolution from 2000 to 2020 are downloaded from the Numerical Terradynamic Simulation Group (NTSG) at the University of Montana (http://www.ntsg.umt.edu (accessed on 1 June 2022)), which has been widely applied to analyze ecosystem services, productivity, and carbon cycles [47,48]. The population data of 100 m spatial resolution are derived from the official WorldPop project website (http://www.worldpop.org.uk (accessed on 1 June 2022)), which is modeled based on the MacDonald Dettwiler and Associates (MDA) GeoCover database and other auxiliary data [49,50]. The land use data of 30 m spatial resolution are obtained for 5-year periods during 2000–2020 from the Resource and Environment Science and Data Center, Chinese Academy of Sciences (http://www.resdc.cn/) (accessed on 1 June 2022) [51,52]. Further, the 30 m resolution land use data are used to calculate the proportion of construction land and cultivated land at 1 km spatial resolution in the BTHUA for each period during 2000–2020 in the study region. 

### 2.3. Method

**Figure 3:**
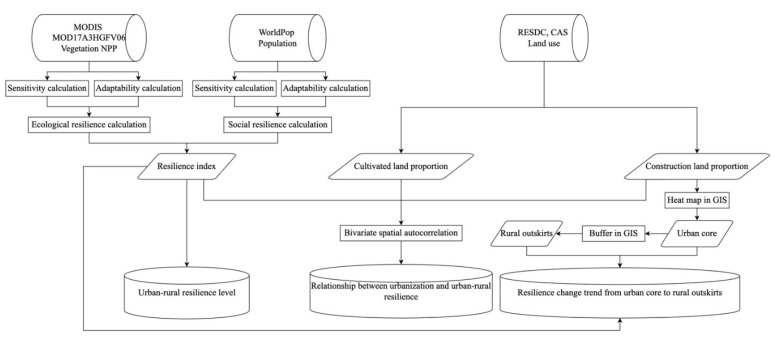
Methodology flowchart of this study.

The overall methodology flowchart of this study is shown in Figure 3. In the resilience assessment, there are both single indicators, also known as resilience surrogates, and composite index evaluation [23]. As an index system demands more data that may not be available on a grid scale, we adopt resilience surrogate evaluation with the evaluation method proposed by Shi et al., which captures the sensitivity and adaptability nature of a resilient system signifying the resistance and recovery processes of resilience. For a detailed calculation method, refer to Shi et al. [35]. In addition, in Shi et al.’s research, NPP data were chosen as the resilience surrogate to characterize natural–physical resilience. Meanwhile, in this research, we also use population data (which is widely used as an urban resilience indicator [21,44,53]) to quantify resilience from the social perspective and come up with the total resilience value of the urban–rural system with equal weights from an ecological and social perspective.

The land use information is extracted to represent the urban–rural system (extract land use). The higher the construction land proportion, the higher the density of the urban area. The lower the construction land proportion, the higher the thickness of the rural area. In exploring the relationship between urbanization (represented by land use change) and urban–rural resilience, we apply the bivariate spatial autocorrelation method [54] to identify the clustering feature of specific land use types (i.e., construction land and cultivated land) with different resilience values. The urban core identification and resilience change trend analysis from the urban core to rural outskirts are performed with heat map and buffer spatial analyst tools in ArcGIS with land use data. Specifically, according to Deng et al. (2008), the urban core is a construction land area contiguous to urban settlements [55]. Each city in the study region has at least one urban settlement. In this study, we identified the most significant urban settlement as the city’s urban core. The benefit of doing this is that it identifies natural cities (the continuous geographic space where human activities gather as a result of urban self-organization processes) [56] instead of administrative cities imposed by the government for the convenience of governance. 

## 3. Urban–Rural Resilience in Beijing-Tianjin-Hebei Urban Agglomeration

**Figure 4:**
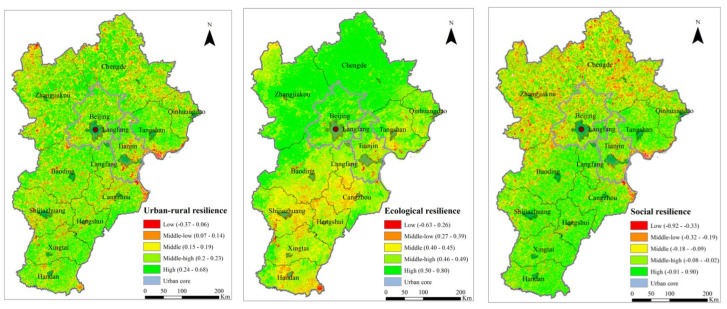
Spatial pattern of urban–rural resilience in the BTHUA.

Figure 4 reveals the results of urban–rural resilience evaluation with resilience from ecological and social dimensions and urban core areas identified in this research. On a regional level, the overall distribution of resilience levels in the region is relatively scattered, with most parts at medium- and high-value resilience levels. The high-value areas are more clustered, and the low-value regions are sporadically distributed. Low-value resilience areas are mainly located in the eastern coastal development area and the northwestern ecological conservation area in the BTHUA region. At the same time, the resilience levels in the central core functional area and southern functional extension area are higher. Overall, the resilience of the eastern coastal region needs to be improved, which is consistent with the low resilience of the coastal areas in previous studies, although the resilience evaluation methods, as well as the study areas, are different [57,58].

When decomposing urban–rural resilience into its ecological and social dimensions, it is found that resilience levels are more aggregated, especially for ecological resilience. The overall trend of ecological resilience in the BTHUA region is high in the northwest and low in the southeast. The eastern coastal areas are also relatively high in ecological resilience, which can be attributed to high forest and grassland coverage generating NPP and is consistent with previous studies [35]. The distribution of ecological resilience is generally uniform with the functional zoning of the BTHUA, with a higher value in the ecological conservation zones. Meanwhile, for social resilience, the trend is quite the opposite of its ecological counterpart, with higher social resilience in the central core functional area and southern functional extension area and lower social resilience in the eastern coastal development area and the northwestern ecological conservation area. In addition, aggregation of higher-social-resilience areas is more prominent, which can be attributed to the population agglomeration phenomenon with the population as the social resilience surrogate. Moreover, the distribution of social resilience is more dispersed than ecological resilience. This can be explained by the distribution of natural-ecological characteristics of the system; for example, vegetation coverage and topographic features are more continuous, while the distribution of social factors, for example, population and infrastructure, is more fragmented.

Regarding urban–rural resilience disparity, ecological and social resilience is high in the urban core areas, while low-resilience grids only exist at the edge of urban cores. Outside the urban core, resilience increases and decreases as the resilience levels are sporadically distributed. In addition, as in some cities, for example, Chengde and Zhangjiakou (mainly serving to preserve ecological functions in the BTHUA), urban centers occupy only a small portion of the city area; it is difficult to analyze the changing trend of resilience from the urban core to rural outskirts from the spatial distribution map. Further numerical analysis of the resilience change along the urban–rural system will be elaborated in Section 4.2. Yet, the size of the urban core area can indicate the urbanization level. In the BTHUA, Beijing and Tianjin’s urban core area accounts for the most significant proportion of the total city area, signifying a high level of urbanization. As for the location of urban cores, in most cities in the BTHUA, the urban core is located at the geometric center of the city, except for Qinhuangdao city (an important port city and coastal open city in China), whose urban core is in its coastal plain area.

The distribution of urban–rural resilience levels at the city level is also relatively dispersed. In the urban core area of Beijing, high-resilience areas are more clustered. The finding is also consistent with previous research in Beijing [59], while in the coastal areas of Tangshan (an important steel production base in China) and Cangzhou (an important petrochemical base and land and sea transportation hub in North China), low-resilience areas are relatively clustered. Coastal regions are more vulnerable to climate change, variability, and rapid urban development and, therefore, have low resilience. For resilience in the two dimensions, from north to south in this region, Zhangjiakou and Chengdu have higher ecological resilience and lower social resilience compared with other cities. Beijing is generally high in ecological resilience, and its social resilience is decreasing from the urban core to the rural outskirts. In Tangshan, Qinhuangdao, and Tianjin, social and ecological resilience patterns are scattered. In Langfang (a city at the very center of the Beijing and Tianjin economic circle), the spatial distribution of social resilience is scattered, and ecological resilience is higher in the north. In Baoding (a regional center city in the BTHUA) and Shijiazhuang (the provincial capital), social resilience is sparsely distributed, and ecological resilience is higher in the west. The same trend goes for Cangzhou and Hengshui (an important railroad transportation hub in the BTHUA), except that ecological resilience is higher in the east. In Xingtai (an important industrial and energy base) and Handan (with a location advantage at the junction of four provinces), social resilience is scattered, and ecological resilience is low in northwest Xingtai, Middle, and southeast Hengshui.

## 4. Land Use Change Impact on Urban–Rural System Resilience

### 4.1. Relationship between Urbanization and Urban–Rural Resilience

**Figure 5:**
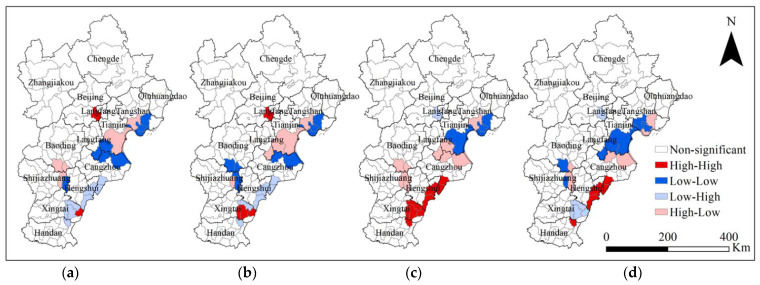
Spatial autocorrelation of urban–rural resilience and (**a**) construction land proportion, (**b**) construction land proportion change, (**c**) cultivated land proportion, and (**d**) cultivated land proportion change in the BTHUA.

To further analyze the relationship between urbanization (signified by cultivated and construction land use change) and urban–rural resilience, a bivariant spatial autocorrelation analysis is carried out at the county level in the BTHUA. The LISA cluster results showed significant high and low spatial concentrations of land use and resilience (Figure 5). From left to right, the two analyzed variables are construction land proportion-resilience (Figure 5a), construction land proportion change-resilience (Figure 5b), cultivated land proportion-resilience (Figure 5c), and cultivated land proportion change-resilience (Figure 5d). The construction land and cultivated land proportion data are the value in 2020, while the proportion change is calculated using the 2020 and 2000 land use data. The High-High legend indicates high land use proportion or its change in specific type and high resilience, and the Low-Low legend indicates a low land use proportion or its change in a certain type and low resilience. The same rule applies to Low-High and High-Low regions. Four major clusters are spotted in the BTHUA, with two high-resilience clusters and two low-resilience clusters.

First, in the Tongzhou district in Beijing, highly urbanized areas with high construction land proportion and low cultivated land proportion are highly resilient. In 2012, the Eleventh Party Congress of Beijing first proposed to make Tongzhou a sub-center of the city. In 2015, the “Outline of Coordinated Development of the Beijing-Tianjin-Hebei Region” designated Tongzhou as the administrative sub-center of Beijing. At present, Tongzhou is the sub-center of Beijing city and hosts the Beijing Municipal People’s Government. Since the proposal of the city’s sub-center, the number of resident populations in Tongzhou district have shown a year-on-year increase with the undertaking and transfer of related industries and the promotion of the policy of population evacuation from the capital. Compared with 2015, the resident population in 2019 increased by nearly 300,000. The region’s urban and rural populations have increased, while the proportion of the urban population in the total population is increasing year by year. By the end of 2021, the urbanization rate of Tongzhou district’s population was 74.3%. There is a threshold in the population carrying capacity of the district, and a continuous and growing population will bring more demands on resources and the environment, which will pose challenges to the city’s resilience. Nevertheless, the Tongzhou sub-center has two major conveniences, the capital and policy support, so it has certain advantages in terms of improved infrastructure, convenient road access, ecological greenery, etc. Investments in agriculture and water services are increasing yearly, enabling it to respond effectively to ecological and environmental risks. As a result, the Tongzhou district currently has a robust comprehensive resilience as part of the urban core in Beijing (Figure 4).

The second major high-resilience cluster covers nine county-level administrative areas in southeast Hebei, including Cangzhou, Hengshui, Xingtai, and Handan. In Cangzhou (Dongguang County, Wuqiao County) and Hengshui (Jing County, Gucheng County), high resilience levels are clustered in rural areas with high-cultivated-land and low-construction-land proportions. In these rural regions, social and ecological resilience are high (Figure 4). For the four counties in Xingtai in this cluster, including Nangong, Guangzong County, Wei County, and Qinghe County, resilience in both urban and rural areas are high in Qinghe. In comparison, the remaining three counties are high in rural resilience. The Qiu County in Handan is also in this high-resilience cluster with low-construction- and high-cultivated-land proportions, indicating high rural resilience. While the first Tongzhou cluster is high in urban resilience, resilience in the second cluster is mainly contributed by rural resilience, where economic development and population clustering are promoted primarily by agriculture and related industries, for example, carton packing machinery in Dongguang, rubber and plastic products in Jing County, the cotton industry in Nangong, and cashmere products in Qinghe.

For major low-resilience clusters, the first covers Tianjin, Tangshan, and Cangzhou. Tianjin, in its major urban districts, covers urban areas (Hebei, Hedong, Hexi, Heping, Nankai, and Hongqiao District), and Dongli, Xiqing, Beichen, Jinnan, and Binhai New District, there is a high level of urbanization with a high construction land proportion and low cultivated land proportion clustered with low resilience. This shows that the resilience of Tianjin’s urban areas needs to be improved. In Jinghai District, a distant suburban county of Tianjin and the north of the major urban districts in Tianjin, the low-construction-land proportion and high-cultivated-land proportion clustered with low resilience, indicating low resilience in rural areas in Jinghai, while, over the study period, the proportion of construction land increased while its cultivated land proportion decreased, indicating rapid land urbanization. However, Jinghai is still in the primary phase of urbanization, and economic and social development is needed to drive urbanization and resilience improvement. Adjacent to the low-resilience clusters in Tianjin, in Tangshan, low-resilience clusters are spotted in north suburban areas, including Luannan County, Fengnan County, and Caofeidian district. In Fengnan County, construction and cultivated land areas are large, indicating low resilience in urban and rural areas. Over the study period, the cultivated land proportion decreased, while resilience remained low. For Luannan County and Caofeidian District, construction and cultivated land areas are low, while the cultivated land proportion increased over the study period. In north Tangshan, urbanization and resilience construction need to be promoted. In addition, in the Tianjin-Tangshan low-resilience cluster, there are rural areas (low-construction-land proportion and high-cultivated-land proportion) in Dacheng, Qingxian, and Huanghua in Cangzhou. According to regional spatial planning, the first major low-resilience cluster is mainly located in the eastern coastal development area in the BTHUA. Resilience, especially rural resilience, needs to be strengthened while developing the port economy.

The second major low-resilience cluster is in the southern functional extension area of the BTHUA, including Dingzhou and Anguo (the largest distribution center for Chinese herbs in China) in Baoding, Shenze County, Jinzhou, and Xinji in Shijiazhuang. Both urban and rural resilience is low in this cluster, except for Xinji, mainly with low rural resilience. Xinji County as a ‘province-managing-county’ is among the top counties in China with a high level of green development, investment potential, technological innovation capability, and new-type urbanization. Dingzhou in this cluster is also among the national pilot cities for new-type urbanization. According to the master plan of BTHUA, in Shijiazhuang and Baoding, the focus is on cultivating high-tech innovative industrial clusters. Nevertheless, this cluster’s resilience along the urban–rural system needs to be improved.

The results in this section show that the relationship between urbanization and resilience is uncertain and that a highly urbanized region or city does not necessarily have high resilience as resilience is still a process that interacts with the natural environment regardless of how developed the society is. In addition, ‘rural’ places are not always low in resilience. Therefore, the urban–rural resilience divide does not abide by homogenous urban (construction land) or rural (cultivated land) dominant areas. Resilience across space can be an active process that also changes along the urban–rural system.

### 4.2. Resilience Change Trend from the Urban Core to Rural Outskirts

**Figure 6:**
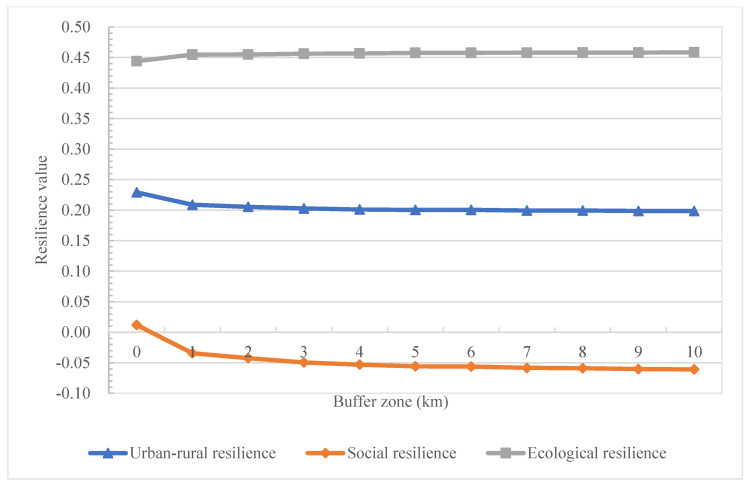
Resilience changes along the urban–rural system with the average value of all cities in the BHTUA.

To further analyze the resilience change along the urban–rural system, we calculate the average resilience value in urban cores and urban buffers from 1 km to 25 km outside the urban cores characterizing rural outskirts. The calculation is performed on both regional and prefectural city scales. The regional average results show that from the urban core to the rural outskirts, there is a general trend that comprehensive resilience decreases with decreased social resilience and increased ecological resilience in this region (Figure 6). This indicates that an urban–rural divide does exist on a regional scale. The study region’s resilience changes significantly from the urban core to the 1 km buffer zone, leveling out at the 2 km buffer zone. For social resilience, such a change is sharper than in ecological resilience. There are apparent urban–rural transitional characteristics in these fringe areas or suburban areas. The gradient of spatial differences between urban and rural elements, landscapes, and functions is significant, and there is a “zone of rapid change” between two types of areas of different natures. At the same time, there are frequent energy and material flows in the fringe areas, where people, materials, technology, and information from urban and rural regions interact and compete.

**Figure 7:**
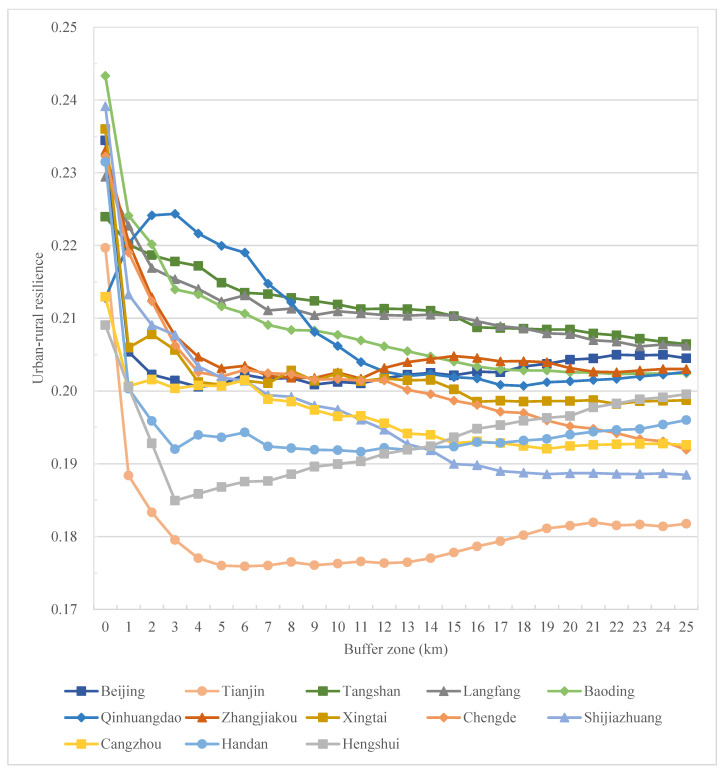
Urban–rural resilience changes along the urban–rural system at the city level in the BHTUA.

**Figure 8:**
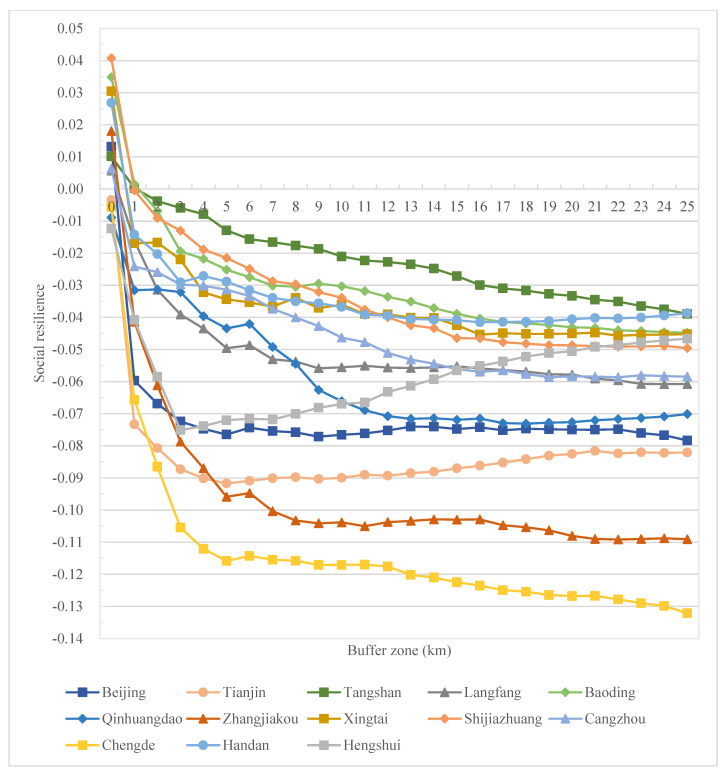
Social resilience changes along the urban–rural system at the city level in the BHTUA.

**Figure 9:**
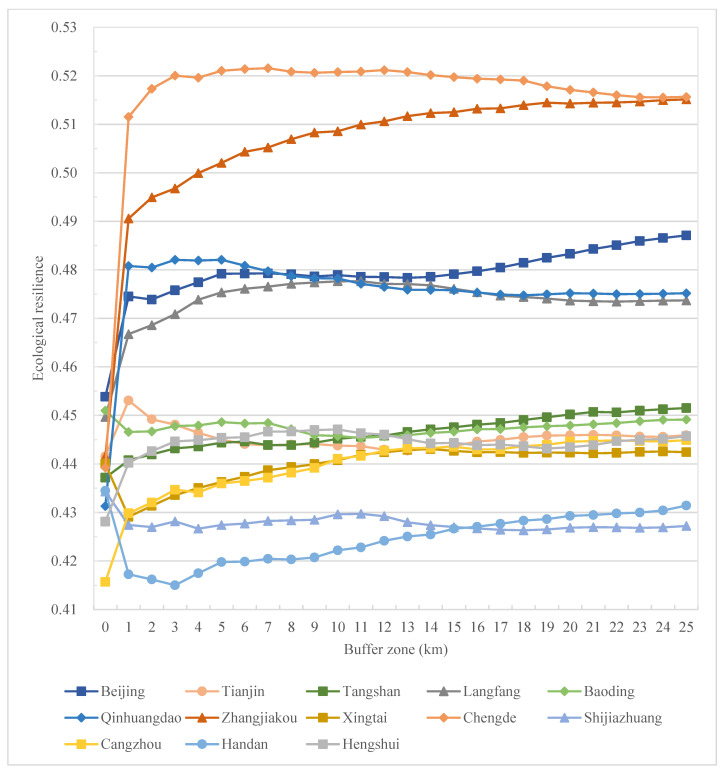
Ecological resilience changes along the urban–rural system at the city level in the BHTUA.

Meanwhile, on the prefectural city scale, generally, all cities reflect a decrease in resilience from the urban core to the rural outskirts, characterizing an urban–rural resilience divide (Figure 7). Combined with the spatial distribution characteristics in Figure 4, with the urbanization process, the resilience level gradually decreases from the urban center to the city’s periphery, and the area with a low resilience level changes from scattered distribution to contiguous distribution. Specifically, at the city level, comprehensive resilience decreases sharply from the urban center to its 3–5 km buffer zone and remains relatively stable in the rural regions (Figure 7). A similar trend goes for social resilience at the city level (Figure 8), while for ecological resilience, it increases sharply from the urban center to its 1–3 km buffer zone and remains relatively stable in the rural regions in this region, except for cities in the west and south of Hebei (Figure 9).

In cities in the BTHUA, though urban cores exhibit high comprehensive resilience, it is not always the highest along the system. For example, in Qinhuangdao, the highest resilience appears in the 3 km buffer zone (Figure 7), and in Hengshui and Tianjin, resilience reaches the bottom in the 3 km and 5 km buffer zone, respectively, and starts to increase, though below the urban peak (Figure 7). This also reveals the regional heterogeneity in urban–rural resilience change along the system. To decompose resilience into its social and ecological dimensions, there are resilience changes in two dimensions with different trends. For social resilience, the trend is relatively consistent among cities in the BHTUA, with a decrease in social resilience away from urban centers, though the degree of reduction varies. From the urban core to the 25 km buffer zone, social resilience reduces significantly in Chengde and Zhangjiakou, while Tangshan experiences the mildest reduction (Figure 8). For the ecological dimension, ecological resilience increases from urban cores to rural outskirts in most cities. While, in Baoding, Shijiazhuang, Handan, and Xingtai, ecological resilience decreases along the urban–rural system, some (i.e., Handan) reach the bottom along the way (Figure 9). 

Although the trend varies between cities and between resilience dimensions, the overall composite resilience of the study area still tends to be weakened from urban to rural. Still, the city and dimension-specific results can be used to give concrete resilience improvement strategies. In addition, results on the grid, county, prefectural city, and regional levels are all generated and visualized in this study to make urban plans at different scales. 

## 5. Discussion

The empirical results in the BTHUA in China reveal the impact of rapid urbanization on urban–rural resilience over the last twenty years. The spatial heterogeneity results could be used for policy reference to make targeted resilience strategies in each region. Moreover, the urban–rural disparity identified in the Beijing-Tianjin-Hebei cluster suggests that China needs to eliminate urban–rural gaps further to realize its new-type urbanization and urban–rural integration policy agendas. Specifically, this region’s spatial distribution of urban–rural resilience is relatively scattered (this is consistent with other research in China, though in different cities [60]), with most regions at medium- and high-value resilience levels. High-resilience areas are more clustered in urban cores, while low-resilience areas are sporadically distributed. For social and ecological resilience dimensions, ecological resilience in the BTHUA region is high in the northwest and low in the southeast. The eastern coastal areas are also relatively high in ecological resilience. For social resilience, its spatial distribution is more scattered than its ecological counterpart and generally higher in the southeast of the BTHUA region.

As for the urban–rural disparity identified in this study, in the BTHUA from the urban core to the rural outskirts, there is a general trend that comprehensive resilience decreases with decreased social resilience and increased ecological resilience. In prefectural cities, generally, all cities reflect an overall decrease in resilience from the urban core to the rural outskirts, though the decreasing trend differs. To further promote the BTHUA integration, the regional divide and the urban–rural divide need to be addressed, which are also emphasized in other land use change studies [61]. The empirical results from this study could serve as a policy reference to identify and visualize low-resilience regions in regional development and make targeted strategies to promote urbanization and resilience enhancement. The resilience results on both the grid and administrative county-level offer fine-scale decision-making reference. Based on this research, we put forward the following policy suggestions. First, rational urban development and population planning reasonably allocate public resources to coordinate economic development, industrial structure upgrading, and ecological environmental protection. Secondly, pay specific attention to the development demand for individual cities and build a regional spatial cooperation mechanism, for example, promote the noncapital function transfer from Beijing to cities such as Baoding or Langfang, and support industrial structure transformation and upgrading in cities such as Tangshan. Finally, human, technological, and capital resources need to be revitalized in this region to promote urbanization and regional integration while enhancing resilience.

## 6. Conclusions

To provide references for regional sustainable development, this study examines urbanization’s social and ecological consequences through the lens of resilience in the urban–rural system with the empirical case of BTHUA in China from 2000 to 2020, contributing theoretically, methodologically, and empirically to urbanization and resilience literature. Theoretically, it contributes to the conceptualization of urban–rural resilience in the urban–rural system, as previous studies examine urban or rural resilience separately. In contrast, this study puts it in the same scope in a metropolitan landscape where an urban–rural system exists. The methodological advantage of this study is that the measure of resilience reflects that resilience is a process rather than a static state. It also reflects the multidimensional composite characteristics of the complex urban–rural system’s natural ecological and social subsystems. In addition, as other urban resilience assessment research was conducted on the administrative district level, this research is conducted on a grid scale to reflect the fine-scale urban–rural disparity.

There are certain limitations of this research, though, with the resilience assessment method that captures the sensitivity and adaptability features of the urban–rural system, over the study period 2000–2020, there is only one resilience value on each grid. Therefore, the evaluation results can only reflect spatial heterogeneity rather than temporal heterogeneity. This assessment method is adequate because this research mainly aims to distinguish the urban–rural disparity in resilience. However, a new resilience assessment method could be developed to further the investigation, preferably to reflect the temporal differences. In doing so, we can try to observe whether there is allometric growth of resilience along urbanization, which could partially explain why resilience is different in different cities across geographical spaces as they might be in different urbanization phases and that the relationship between urbanization and resilience can be either linear, sublinear, or even super-linear indicated in the urban scaling laws [62]. This also constitutes our future research agenda. In addition, sustainable development commonly embraces three essential aspects: environment, economy, and society. The resilience index in this study mainly captures environmental and social features. As for the economic aspect, in the preliminary data analysis, we use GDP data to quantify economic resilience and find that it is similar to social resilience calculated with population data. This also shows that social and economic development is homogeneous in the study region. Therefore, in the analysis of this paper, we exclude the economic aspect, while for future studies in other areas, the resilience index that captures the three dimensions of sustainable development could be explored to characterize sustainability with resilience better.

## Data Availability

All data and materials are available upon request.
